# Supplementary figures and images for: Semaphorin 3E deficiency dysregulates dendritic cell functions: In vitro and in vivo evidence
Source: PLoS One. 2021 Jun 29;16(6):e0252868. doi: 10.1371/journal.pone.0252868 (PMC8241044; doi:10.1371/journal.pone.0252868)

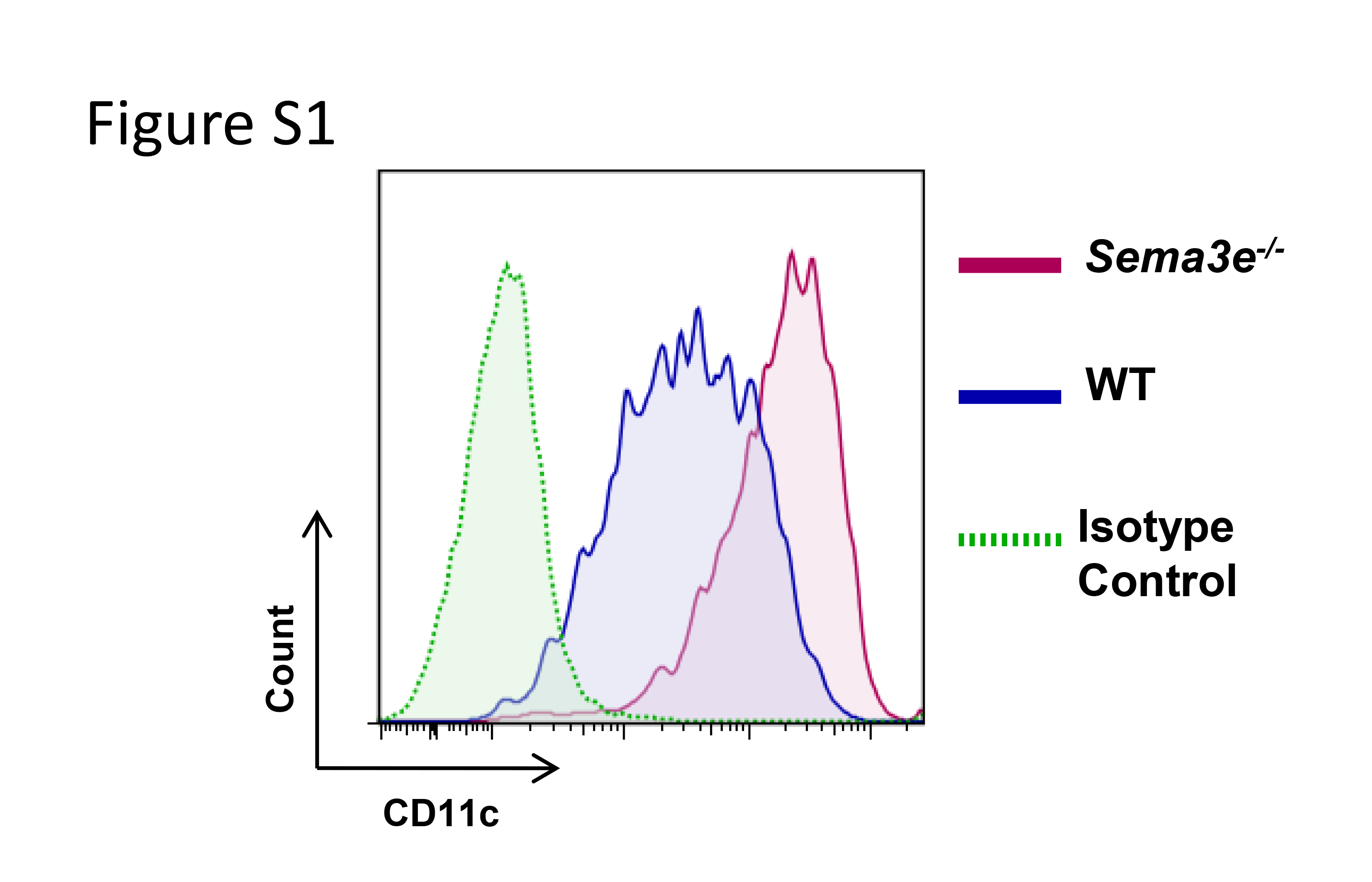

Supplement: S1 Fig — (TIF) [file pone.0252868.s001.tif]

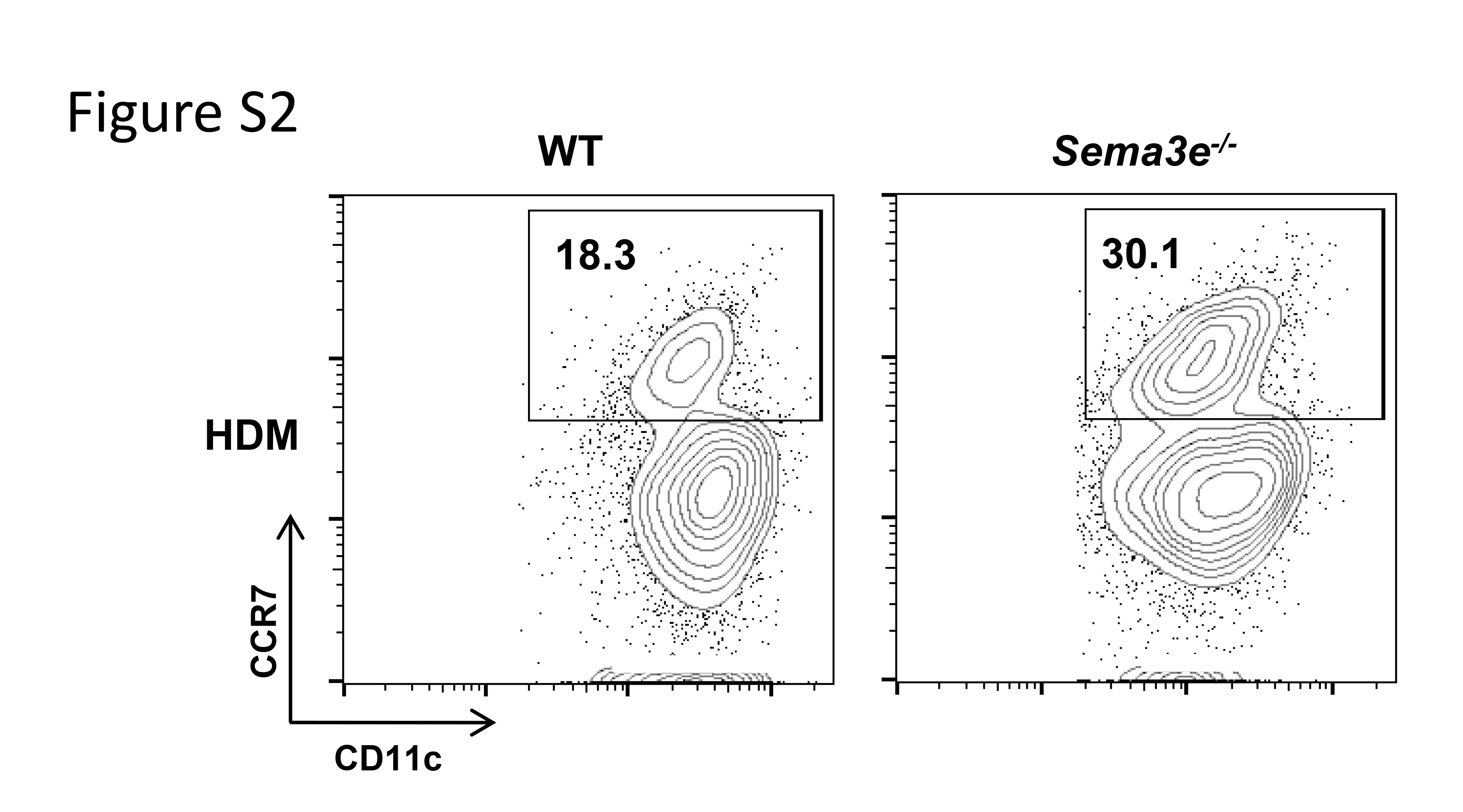

Supplement: S2 Fig — (TIF) [file pone.0252868.s002.tif]

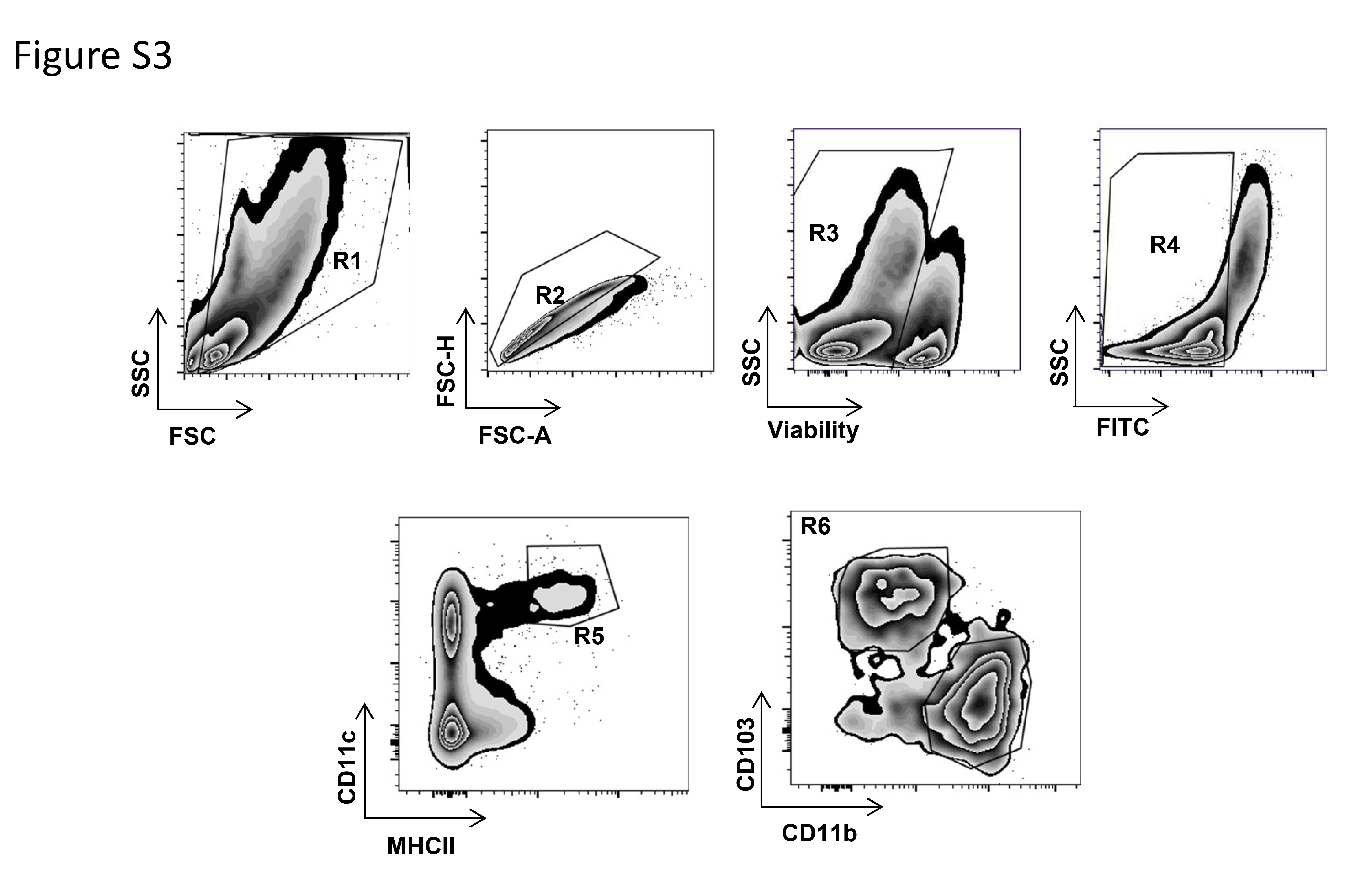

Supplement: S3 Fig — In total cell population (R1), single (R2) live (R3) cells were selected and macrophages were excluded (R4). Then, total pulmonary cDC were determined based on high surface expression of MHCII and positivity for CD11c (R5). Finally, distinct CD11b vs CD103 expressing cDC were characterized (R6). (TIF) [file pone.0252868.s003.tif]
