# Supplementary material for: Semaphorin 3E deficiency dysregulates dendritic cell functions: In vitro and in vivo evidence
Source: PLoS One. 2021 Jun 29;16(6):e0252868. doi: 10.1371/journal.pone.0252868 (PMC8241044; doi:10.1371/journal.pone.0252868)
Supplement: Attachment — Submitted filename: Response to Reviewers.docx [file pone.0252868.s004.docx]

**Reviewer #1)**

1. Author have used in vitro BMDCs model to show the migratory defect in Sema3E deficient BMDCs compared to Sema3E sufficient one. The data showing increased CCR7 expression in Sema3E deficient BMDCs in presence of CCL21 looks promising. However, flow data presented is not convincing as basal CD11c expression in Sema3E deficient BMDCs seems high while after adding CCL21 it comes to normal. Author should also include the data with alternative ligand CCL19 which could add to conclusion that CCL21 and CCL19 both lead to the CCR7 pathway activation controlling the migration of DCs.

Reply: Thank you for your comment. As presented in Fig. 1B, the percentage of CCR7 expressing CD11c+ cells from WT mice have been increased from 5% at the baseline to 9% after stimulation with CCL21. As a novel finding we have shown that genetic deletion of Sema3E increased the baseline expression of CCR7 which explains why BMDC from KO mice have an increased basal migration (Fig.1A). This phenotype sustained after CCL21 stimulation. We have observed that stimulation of BMDC from Sema3E KO mice with CCL19 induces cell migration more robustly than those of WT controls. Unfortunately, we have not investigated whether CCL19 would similarly signal as of CCL21.

2. After sensitization with HDM-APC it is clearly shown the increased uptake of allergen by lung CD11b+cDCs of Sema3E deficient mice. It would better idea to add the HDM-APC+ CD11b+cDCs data from mediastinal lymph node with same experiment. Also, it is not mentioned how many mice were used to perform this experiment.

Reply: Thank you for your comment. There is a technical issue regarding the low number of this specific cell subset in the MLN post-sensitization. In fact, the size of lymph nodes is very small 3 days after exposure to one dose of HDM which makes the FACS isolation of these cells (pre-gated as explained in the manuscript) quite challenging.

3. Figure 3, the representative flow plot for gating strategy do not add to results, it could be moved to supplemental data. What is FITC in SSC vs FITC? Is that representing SiglecF?

Reply: Thank you for your comment. We have removed the gating strategy and presented it as a supplemental figure in the revised manuscript. FITC is a dumb channel to exclude autofluorescent macrophages.

4. Increased expression level of PD-L2 and IRF-4 in lung cDCs seems very promising. What are the numbers representing? Is the data from a whole lung?

Reply: Thank you for your comment. The numbers represent the absolute counts of corresponding cell subsets from the total live whole lung cells.

5. Apart from CCR7, what was the expression level of other activation markers in the DCs during the sensitization phase? Did author check for activation status of maturation markers in the DCs from Sema3E deficient mice?

Reply: Thank you for your comment. As a hypothesis-driven approach to address the mechanism underlying increased DC migration in the absence of Sema3E, we specifically focused on CCR7 as an essential receptor in migration of dendritic cells and homing in the lungs.

6. Figure1B, flow plots are not uniform that needs to be replaced.

Reply: Thank you for your comment. We double checked the flow plots in Figure 1B. We think the main reason they do not look uniform is the impact of Sema3E deletion as well as stimulation with CCL21 which directly affect shifting the Y-axis upward indicating upregulation of CCR7.

7. Figure2A, HDM amount (100 ug) contradicts the materials methods (page7, line 126, 1 ug).

8. Page7, line 116, clone 418 should be N418.

9. Page2: Line 29, replace ‘time’ with ‘type’.

10. Page5: Line 80, replace ‘BMDC’ with ‘BMDCs’.

11. Page14: Line 273, OF should be corrected in the revised version.

Reply: Sorry for the typos. We have corrected all of them in the revised version as highlighted in yellow.

**Reviewer #2)**

This research has merit as it will help in development of novel immunotherapeutic strategies for the treatment of allergen based diseases in which immune regulation is impaired. Authors have presented the data clearly and have conducted all the necessary assays and statistical analysis.

Reply: Thank you very much.
